# Use of web and mobile device technologies in the management of childhood asthma: a systematic review and meta-analysis

**DOI:** 10.1016/j.jped.2025.01.013

**Published:** 2025-04-09

**Authors:** Maria Gabriella Adeodato Prado, Francisco Placido Nogueira Arcanjo, Luiz Odorico Monteiro de Andrade, Ivana Cristina de Holanda Cunha Barreto, Lizandro de Andrade Teles, Jeferson de Sousa Justino, Edcley de Souza Teixeira, Marya Clara Barros Mororó

**Affiliations:** a Universidade Federal do Ceará, Programa de Pós-Graduação em Ciências da Saúde, Sobral, CE, Brazil; b Fundação Oswaldo Cruz, Eusébio, CE, Brazil; c Universidade Federal do Ceará, Faculdade de Medicina, Sobral, CE, Brazil

**Keywords:** Asthma, Child, Child health, Wireless technology

## Abstract

**Objective:**

The objective of this review is to assess the use of support tools for children with asthma, based on web and mobile device technologies, and their impact on asthma control.

**Method:**

This is a systematic review conducted in accordance with the PRISMA guidelines and the Joanna Briggs Institute (JBI) Manual. The research question defined by the PICO strategy was: ‘‘What are the effects of web-based and mobile device support tools on asthma control in children?’’ The search was conducted in the Medline (via PubMed), SciELO, and Embase databases between October and December 2023, with completion in July 2024.

**Results:**

The systematic review analyzed 388 articles and selected 4 studies on technologies for managing asthma in children. The studies showed that mobile apps and electronic monitoring improve asthma control, treatment adherence, and caregivers’ quality of life. The meta-analysis showed a mean increase in Asthma Control Test (ACT) scores of 2.73 (95 % CI: 1.95, 3.51) with *P* < 0.0001, indicating a significant improvement in asthma control scores, highlighting the effectiveness of these technologies.

**Conclusions:**

This study demonstrates that digital tools, such as web technologies and mobile devices, can significantly improve the management of childhood asthma, as reflected by an increase in Asthma Control Test (ACT) scores. Despite limitations, the findings are promising. Future research is needed to strengthen the evidence and guide clinical practice in pediatric asthma management.

## Introduction

Asthma is a chronic inflammatory airway disease that affects millions of children worldwide, characterized by a wide spectrum of symptoms ranging from mild manifestations to severe clinical cases, substantially impacting the respiratory system and, consequently, the quality of life of patients [1]. The prevalence of childhood asthma varies globally, but it is estimated to affect about 10 % of school-aged children, being one of the leading causes of hospitalization, school absenteeism, and reduced quality of life in children.^2^

The search for new approaches to asthma management has intensified, particularly those incorporating digital technologies. The integration of web-based and mobile device technologies into the care of childhood asthma has shown promising potential. These tools offer new ways to monitor symptoms, educate patients and caregivers, and promote more effective communication between healthcare professionals and families.^3^

The use of mobile and web technologies in asthma management not only facilitates access to critical information about the disease but also enables continuous and adaptive monitoring. Wearable devices and health apps can record and analyze data on the frequency and intensity of symptoms, medication use, and exposure to environmental factors. When integrated with telemedicine platforms, this data can be shared with healthcare professionals in real-time, allowing for precise treatment adjustments and a quicker response in crisis situations.^4^

These technologies can play a key role in educating patients and caregivers, empowering them to better manage the disease. Educational programs integrated into mobile apps can enhance patients’ understanding of their condition, increase treatment adherence, and reduce anxiety associated with asthma management.^5^ Telemedicine also emerges as an important tool, allowing for remote consultations that are especially useful for monitoring disease progression and adjusting treatments without the need for frequent office visits, which is particularly advantageous for families in remote areas or with limited access to specialized services.^6^

Despite the potential of these technologies, it is important that they are evaluated systematically and rigorously to ensure their effectiveness and usability. The diversity of available digital tools and the variation in the quality of evidence associated with them highlights the need for a systematic review that consolidates existing knowledge and identifies best practices for managing and controlling childhood asthma.

This review evaluates the available evidence, identifies knowledge gaps, and provides evidence-based guidelines for clinical practice, with the aim of assessing the use of support tools for children with asthma based on web and mobile device technologies, and analyzing their impact on asthma control.

## Methods

This is a systematic review conducted based on the PRISMA (Preferred Reporting Items for Systematic Reviews and Meta-Analyses) guidelines and the Joanna Briggs Institute (JBI) Manual.^7^^,^^8^ The protocol for this study was registered in PROSPERO with registration number CRD42024554735.

The research question was developed according to the ‘‘PICO’’ strategy, defined as: ‘‘What are the effects of web-based and mobile device support tools on asthma management in children?’’.^7^

The inclusion criteria for this study involved various aspects to ensure the relevance and quality of the data analyzed. Observational studies, such as cohort and case-control studies, as well as randomized clinical trials investigating the use of web technologies and mobile devices in asthma management in children, were included. The target population consisted exclusively of children diagnosed with asthma, with no restrictions regarding gender, ethnicity, or severity of the disease, covering the age range from 0 to 18 years. The considered interventions included the use of web-based tools and mobile devices, such as apps, educational websites, remote monitoring platforms, and interactive games. Studies reporting relevant outcomes for asthma management, such as treatment adherence, frequency of exacerbations, symptom control, emergency department visits, health-related quality of life, and user satisfaction with technology, were evaluated. There were no restrictions on the language or publication date of the studies.

On the other hand, studies that did not have empirical data, such as conference abstracts, editorials, comments, letters, and technical reports, were excluded from the analysis. Studies that did not specify the age range of the participants were also excluded to ensure a pediatric focus. Studies with flawed methodology or high risk of bias were likewise excluded to ensure the integrity and quality of the data analyzed.

The search took place between October and December 2023 and was reviewed and completed in July 2024, using the databases Medline via PubMed (https://pubmed.ncbi.nlm.nih.gov/), SciELO (https://www.scielo.br/), and Embase (https://www.embase.com/). The search utilized indexing terms (MeSH in the PubMed database and EMTREE in the Embase database) and synonyms related to the population (Asthma, Bronchial Asthma, Asthma, Bronchial, Child, Children), the intervention (Application, Mobile). A clinical trial filter^9^ was used in combination with the MeSH terms already described: (randomized controlled trial[pt] OR controlled clinical trial[pt] OR randomized controlled trials[mh] OR random allocation[mh] OR double-blind method[mh] OR single-blind method[mh] OR clinical trial[pt] OR clinical trials[mh] OR (“clinical trial”[tw]) OR ((singl*[tw] OR double*[tw] OR trebl*[tw] OR tripl*[tw]) AND (mask*[tw] OR blind*[tw])) OR (“latinsquare”[tw]) OR placebos[mh] OR placebo*[tw] OR random*[tw] OR research design[mh] OR follow-up studies[mh] OR prospective studies[mh] OR cross-over studies[mh] OR control*[tw] OR prospectiv*[tw] OR volunteer*[tw]) NOT (animal[mh] NOT human[mh]). A supplementary manual search was also conducted.

The critical analysis of the studies included in the systematic review was conducted independently by two reviewers, using specific tools for each type of study. Randomized clinical trials were assessed using the RoB 2.0 tool, while before-and-after studies were evaluated with the NIH Quality Assessment Tool.^10^^,^^11^ Data synthesis was conducted both qualitatively, with results presented in tables and charts, and quantitatively, through meta-analysis using Review Manager software (version 5.4). Heterogeneity was assessed using the I² test, and the results of the meta-analysis were presented in forest plots.

## Results

**Figure 1:**
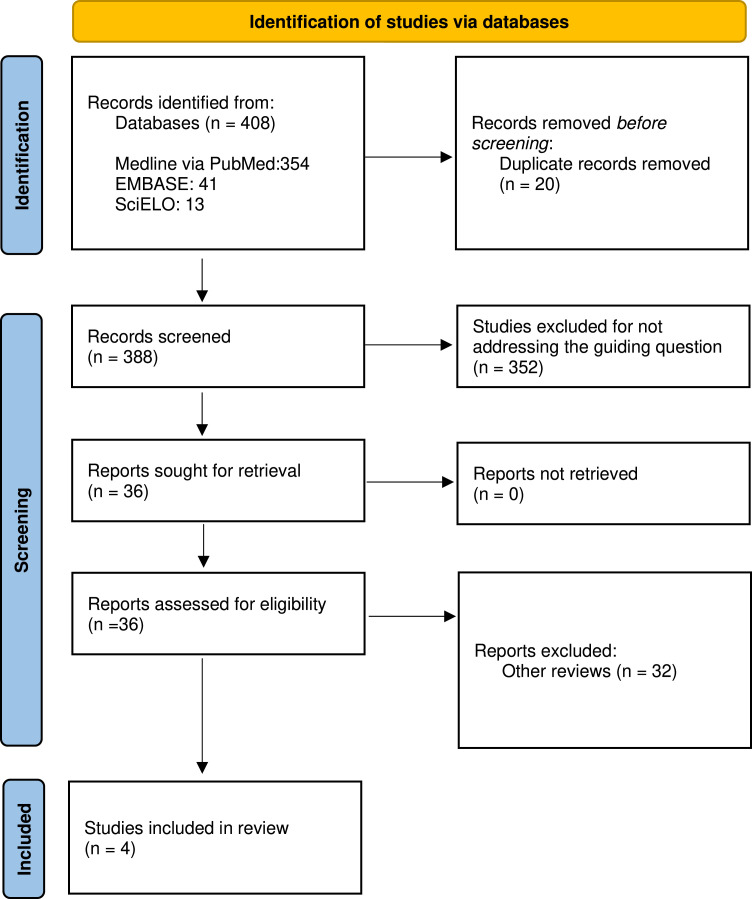
Flowchart of study selection according to PRISMA.

The systematic search identified 354 studies in Medline via PubMed, 41 in Embase, and 13 in SciELO. After the removal of 20 duplicates using Rayyan software, 388 articles were analyzed based on their titles and abstracts. Following the analysis, 384 studies were excluded for not meeting the established criteria: 352 for having divergent topics and 32 for being literature reviews, leaving 4 studies for analysis. These 4 studies were evaluated in full for eligibility for inclusion in the systematic review. The selection process followed the PRISMA flow diagram (Figure 1). Figure 1 represents the flowchart of the selection process for the articles included in the systematic review, detailing the identification, screening, and inclusion stages.

Table 1 presents the main characteristics of the studies included in this systematic review.

**Table 1:** Description of the studies included in the systematic review.

Title	Author	Objective	Method	Results	Limitations	Response to the problem	Population
Mobile-based asthma action plans for adolescents	Burbank et al. [12]	The primary purpose of the study was to examine the feasibility and use of a mobile asthma action plan (AAP) among adolescents with persistent asthma, aiming to understand how this tool could integrate into their daily routines and impact asthma self-management.	The study adopted a single-arm design, exploring the implementation of an asthma action plan (AAP) via a mobile app in adolescents aged 12 to 17 with persistent asthma. Participants had their personalized AAP made available on a smartphone app and were encouraged to record daily symptoms or peak flow measurements, receiving immediate feedback based on their AAP instructions. App usage, participant satisfaction, and the effects of app use on self-efficacy scores and asthma control were analyzed.	The adolescents used the mobile AAP a mean of 4.3 days per week, and satisfaction with the tool was high. A significant improvement was observed in asthma control scores (ACT) and self-efficacy for preventing asthma attacks, particularly among participants with uncontrolled asthma at the start of the study.	The study was limited by its short observation period (8 weeks) and single-arm design, which may restrict the generalizability of the results. Additionally, the sample of participants was relatively small, and most of the data collected were based on self-reports.	The results suggest that mobile AAPs are a feasible methodology for communicating AAP instructions to adolescents, potentially improving asthma control and self-efficacy in asthma management. The adolescents’ acceptance and frequent use of the app indicate that mobile technologies can be a valuable tool in asthma self-management for this population, especially in rural areas and those with limited access to healthcare resources.	The mean age was 13.5 years, with a standard deviation of 3.47. It is noteworthy that there were 20 participants at the pre-intervention stage, but 2 participants were lost by the post-intervention stage (*n* = 18).
Electronic Adherence Monitoring in a High-Utilizing Pediatric Asthma Cohort: A Feasibility Study	Kenyon et al. [13]	To evaluate the feasibility and acceptability of an electronic treatment adherence monitoring intervention delivered by a community health worker to high-risk children with asthma in an urban environment.	A prospective pilot cohort study was conducted involving children with moderate to severe persistent asthma, using electronic monitoring devices attached to their inhalers. The intervention, lasting 3 (three) months, included motivational interviews conducted by a specialized community health worker and electronic monitoring of the use of controller and rescue medications. The devices recorded inhaler usage data and alerted for inappropriate medication use, allowing the community health worker to contact families as needed to support treatment adherence.	Three distinct patterns of controller use were identified at baseline: sustained use, periodic use, and discontinued use. All participants began using the electronic devices, but there were issues with data transmission and loss of devices. Most caregivers who completed the survey considered the technology acceptable, and there was a mean improvement in ACT scores.	The study had a small sample size and was limited to a single clinic, which may not reflect the diversity of experiences in other contexts. Additionally, there were significant challenges related to the maintenance and data transmission of the electronic monitoring devices, which affected the consistent data collection throughout the study.	The study demonstrated that electronic adherence monitoring in a high-risk pediatric population is feasible and generally well accepted, but it faces significant challenges that need to be addressed to improve the effectiveness of these interventions in supporting children with asthma.	The study had 14 participants, with ages ranging from 3 to 9 years and a median age of 3.5. Approximately 57 % were male and about 43 % were female.
Developing and evaluating ASTHMAXcel adventures: A novel gamified mobile application for pediatric patients with asthma	Hsia et al. [14]	To evaluate the impact of ASTHMAXcel Adventures, a gamified pediatric version based on the guidelines of the ASTHMAXcel mobile app, on asthma control, knowledge, healthcare utilization, and patient satisfaction.	The study was a prospective single-arm study that included pediatric patients with asthma who received asthma education through the ASTHMAXcel Adventures mobile app on an iPad tablet on-site. Instruments such as the ACT, AIRS-SR, PAIS, and Customer Satisfaction Questionnaire-8 were used to assess asthma control, knowledge, patient satisfaction, and healthcare utilization. The study duration was not explicitly specified, but according to the date descriptions, it lasted approximately 14 months.	An increase was observed in the proportion of patients with controlled asthma, improvements in asthma knowledge and quality of life, and a reduction in emergency department visits and oral prednisone use. Patient satisfaction with the app was high, with a mean score on the Customer Satisfaction Questionnaire of approximately 30 out of 32 across all visits.	The study had a relatively small sample size and was conducted at a single center, which may limit the generalizability of the results. Additionally, the intervention was offered only in English, which may restrict its applicability to non-English-speaking populations.	The ASTHMAXcel Adventures app proved to be an effective and well-accepted tool for improving asthma control, disease knowledge, quality of life, and reducing the use of emergency services in pediatric patients with asthma. It holds significant potential to be integrated as a supportive tool in the management of pediatric asthma.	The study comprised 39 participants, with a mean age of 10.5 years and a standard deviation of 2.6. Approximately 51.28 % were male and about 48.72 % were female.
Sensor-Based Electronic Monitoring for Asthma: A Randomized Controlled Trial	Gupta et al. [15]	Determine the effectiveness of a clinically integrated sensor-based inhaler monitoring intervention in improving asthma symptom control and asthma-related outcomes among a diverse sample of children with moderate to severe asthma.	Caregiver-child dyads were randomized to receive inhalation sensors that allowed for electronic monitoring of medications. Outcomes included scores on the Asthma Control Test (>19 indicated controlled asthma) and asthma-related healthcare utilization. The caregiver's quality of life and the child's adherence to ICS were also assessed. The study lasted 12 months, with evaluations at 1, 3, 6, 9, and 12 months.	The dyads were assigned to the control or intervention arms. At the final assessment, the mean score on the Asthma Control Test increased from 19.1 (SE = 0.3) to 21.8 (SE = 0.4) in the intervention group and from 19.4 (SE = 0.3) to 19.9 (SE = 0.4) in the control group (Δ intervention-control = 2.2; SE = 0.6; *P* < 0.01). The adjusted rates of emergency department visits and hospitalizations in the intervention group were significantly higher (incidence rate ratio emergency department = 2.2; SE = 0.5; *P* < 0.01; incidence rate ratio hospital = 3.4; SE = 1.4; *P* < 0.01) at the final assessment compared to the control group. The caregiver's quality of life was higher in the intervention group at the end (Δ intervention-control = 0.3; SE = 0.2; *P* = 0.1) than in the control group.	Some inhalers were not compatible with the sensor, requiring participants to manually enter data into the app. Only the participants in the intervention group received sensors, preventing comparisons between groups regarding adherence to ICS or use of SABA. Although the study attempted to comprehensively capture healthcare utilization, some events may not have been recorded. Generalizability is limited for non-English-speaking individuals, as they were excluded due to the lack of an app in other languages. Additionally, there were missing data due to incomplete responses or sensor failures over time.	The results suggest that sensor-based inhaler monitoring with clinical feedback may improve asthma control and the caregiver's quality of life across diverse populations. Increased healthcare utilization was observed among participants in the intervention group compared to the control group, indicating that further refinement is needed.	The study segmented participants into two groups, one intervention group (*n* = 125) and one control group (*n* = 127), totaling 252 participants. The mean ages of the intervention and control groups were 9.3 (SD = 3.2) and 9.2 (SD = 3.5), respectively. The distribution by biological sex was as follows: male (69.3 % vs. 63.2 %) and female (30.7 % vs. 36.8 %).

ACT, teste de controle da asma; PAIS, Pediatric Asthma Impact Survey; AIRS-SR, Asthma illness representation scale self-report; SE, standard error; ICS, inhaled corticosteroids; SABA, short-acting beta-agonists; “n”, number; “int”, intervention; “cont”, control.

The assessment of the methodological quality of the included studies was analyzed using specific tools for each study design. For randomized clinical trials, the RoB 2 tool was used. For before-and-after studies, the NIH Quality Assessment Tool for Before-After (Pre-Post) Studies with No Control Group was utilized.

Table 2 provides an analysis of the quality of observational studies according to the NIH Quality Assessment Tool.

**Table 2:** Assessment of risk of bias in cohort and cross-sectional studies according to the NIH Quality Assessment Tool.

Criterion	Burbank et al. [12]	Kenyon et al. [15]	Hsia et al. [14]
1. Was the research objective clearly stated?	Yes	Yes	Yes
2. Was the study population clearly specified and defined?	Yes	Yes	Yes
3. Was the participation rate of eligible individuals at least 50 %?	Yes	No	Yes
4. Were all subjects recruited from the same or similar populations (including the same time period)?	Yes	Yes	Yes
5. Was a justification provided for the sample size, including a power description or effect size estimate?	No	No	No
6. Were the exposures of interest measured before the outcomes?	Yes	Yes	Yes
7. Was the time period sufficient to observe an association between the exposure and the outcome of interest?	Yes	Yes	Yes
8. Were different levels of exposure examined?	Not applicable	Not applicable	Not applicable
9. Were the exposure variables clearly defined, valid, reliable, and applied consistently?	Yes	Yes	Yes
10. Were the exposures assessed more than once over time?	Yes	Yes	Yes
11. Were the outcome variables clearly defined, valid, and applied consistently?	Yes	Yes	Yes
12. Were the outcome assessors blinded to the participants’ exposure status?	No	No	No
13. Was the loss to follow-up after baseline 20 % or less?	Yes	No	No
14. Were the main confounding variables measured and statistically adjusted for their impact?	No	No	Yes
Overall quality	**Moderate**	**Moderate**	**Good**

**Figure 2:**
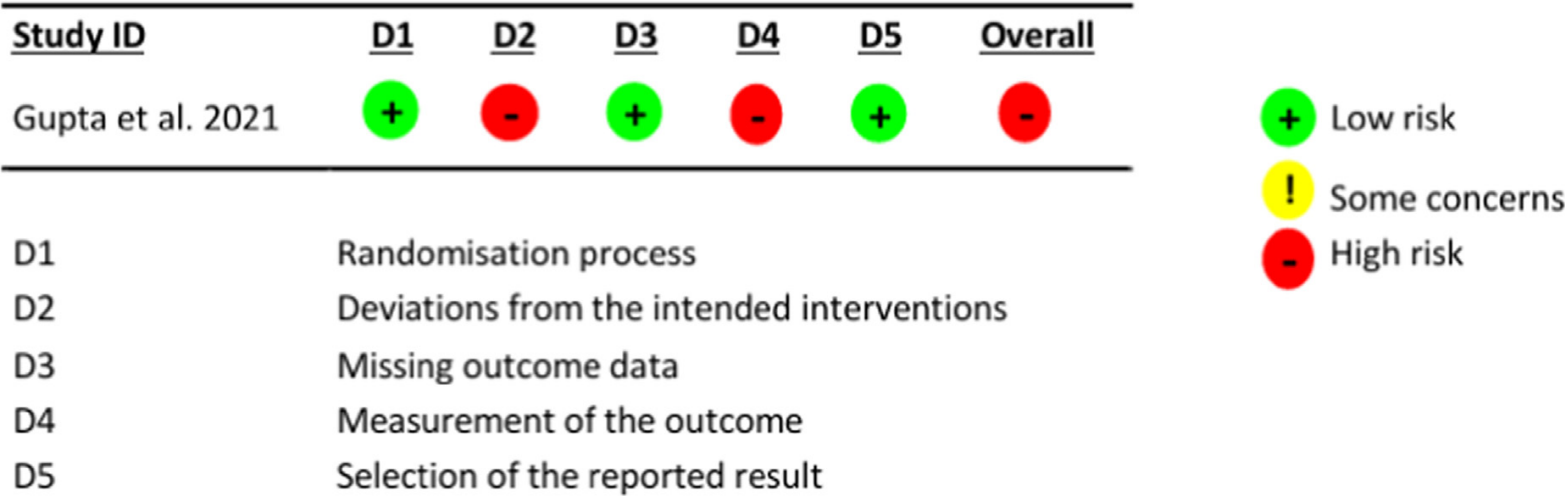
Assessment of the risk of bias in the study by Gupta et al.^13^

Figure 2 describes the assessment of the study by Gupta et al.^13^ for the outcome of asthma control, using the RoB 2.0 tool.

The assessed domains were as follows: 1) D1, bias in the randomization process: this concerns the method used to generate the allocation sequence of participants, which should be random; the method used to allocate participants to the study groups; and evaluates whether there were imbalances in participant characteristics suggesting some issue with the randomization process; 2) D2, deviations from the intended intervention: this concerns whether the patient and study team were unaware (blinded) of which group the patient was allocated to and whether there were deviations from the proposed intervention that could affect the outcome; 3) D3, bias due to missing data: this concerns loss to follow-up of study participants and, in the case of losses, the reason for their occurrence; 4) D4, bias in outcome assessment: this concerns whether the outcome assessors (patient, researcher, or evaluator) were unaware of which group participants were allocated to, considering outcomes that could be affected by knowledge of the intervention received; and 5) D5, bias in reporting outcomes: this concerns the possibility that the authors may have assessed outcomes through multiple evaluations but reported only the most convenient one.

The management and control of childhood asthma showed significant improvements in the evaluated studies. Burbank et al.^12^ investigated the use of mobile device-based Asthma Action Plans (AAP), which allowed adolescents to better manage their condition through a smartphone app. The results indicated a significant improvement in asthma control and user self-efficacy, with high acceptance and frequency of use, especially among those with uncontrolled asthma at the beginning of the study. This study highlights the potential of mobile apps as effective tools for self-management of asthma in young populations.

Kenyon et al.^13^ explored the feasibility of electronic monitoring of treatment adherence in high-risk children with asthma. Despite technical challenges, such as data transmission issues and loss of devices, the technology was well received and resulted in improvements in asthma control. Another advancement was observed by Hsia et al.^14^ who evaluated the gamified app ASTHMAXcel Adventures, which proved effective in improving asthma knowledge, disease control, and reducing the use of emergency services. Finally, Gupta et al.^15^ demonstrated that sensor-based inhaler monitoring can significantly improve asthma symptom control and the quality of life of caregivers, although further refinements are needed to optimize the effectiveness of this technology.

In the study by Hsia et al.^14^ there were limitations related to the lack of blinding of outcome assessors and the high loss to follow-up rate, which are associated with a risk of measurement bias. Additionally, it was not possible to determine whether all eligible patients were screened and included in the study, which may impact selection bias if not conducted appropriately.

In the study by Burbank et al.^12^, there were limitations related to the lack of blinding of outcome assessors, which is associated with a risk of measurement bias. Additionally, it was not possible to determine whether all eligible patients were screened and included in the study, which may impact selection bias if not conducted appropriately. A sample size calculation was also not performed, which is associated with sampling bias.

In the study by Kenyon et al.^13^ there were limitations related to the lack of blinding of outcome assessors, which is associated with a risk of measurement bias. Additionally, it was not possible to determine whether all eligible patients were screened and included in the study, which may impact selection bias if not conducted appropriately. A sample size calculation was also not performed, which is associated with sampling bias.

**Figure 3:**
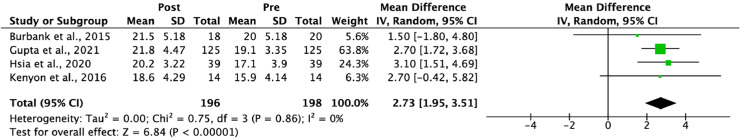
Meta-analysis of the asthma control outcome assessment through the mean difference of the Asthma Control Test (ACT). CI, confidence interval; SD, standard deviation.

The meta-analysis conducted included four studies that evaluated the effects of support tools based on web technologies and mobile devices for children with asthma. These studies assessed asthma control using the Asthma Control Test (ACT). There was a mean increase in ACT scores before and after the interventions of 2.73 (95 % CI: 1.95, 3.51), indicating a significant improvement in clinical outcomes following the intervention. The absence of overlap of the confidence interval with zero reinforces the statistically significant efficacy of the support tools (Figure 3).

There was no statistical heterogeneity among the studies, as indicated by an I² value of 0 %, suggesting that the study results are consistent, which may strengthen the validity of the findings and the reliability of the conclusions regarding the efficacy of the technological interventions.

## Discussion

Overall, the studies showed consistent results, indicating better asthma control following the use of web-based technologies and mobile devices.

In addition to the direct benefits for children, digital technologies can also positively influence the quality of life of caregivers. The use of mobile applications allows for more consistent monitoring of asthma, which can reduce anxiety and stress associated with daily care.^15^ Proper asthma control can significantly impact the quality of life of patients and their caregivers, with a strong correlation shown between asthma control scores and caregiver quality of life.^16^ Factors such as continuous use of medications, treatment adherence, asthma monitoring, and inhalation techniques are fundamental pillars of asthma control.^17^

Specifically, the clinical trial by Gupta et al.^15^ reported a mean increase of 2.7 points in ACT scores among patients who used the intervention compared to the control group after 12 months of intervention. Additionally, the study by Hsia et al.^14^ also showed significant improvements in these scores when comparing patients before and after using the intervention, with mean differences of 3.10 (95 % CI: 1.51, 4.69).

In contrast, the studies by Kenyon et al.^13^ and Burbank et al.^12^ showed positive results but without statistical significance. This may be explained by the small sample sizes (14 and 18 participants, respectively), which reduced the power of the studies. On the other hand, Stukus et al.^18^, when evaluating a different outcome, such as the number of emergency room visits, also did not observe significant reductions after the use of a mobile application. However, the authors suggest that the effectiveness of this intervention may depend on factors such as adherence to using the app and the demographic characteristics of the target population, which may also have influenced the results of Kenyon et al.^13^ and Burbank et al.^12^

The use of an application or digital tool in asthma leads to the personalization of digital interventions, which can have variable effectiveness depending on individual patient characteristics, such as age, severity of asthma, and familiarity with technology.^18^, ^19^, ^20^

The overall combined effect obtained from the meta-analysis reinforces the conclusion that support tools based on web technologies and mobile devices are effective in providing significant improvements in the management of childhood asthma. By combining the four studies, a significant mean increase of 2.73 points in the ACT (95 % CI: 1.95–3.51) was observed after the use of the intervention, demonstrating the robustness of these interventions in improving clinical outcomes in children with asthma.

The preference of adolescents for asthma action plans based on smartphones, as demonstrated by Perry et al.^21^, supports the potential efficacy of technological interventions that utilize mobile devices for asthma management. The recognition of the effectiveness of digital interventions in improving treatment adherence in asthma can be promising for better control.^22^ These findings align with the results of this review, which also highlight the acceptance and benefits of these technological tools in the management of childhood asthma.

Monitoring asthma control is essential for adjusting treatment and tracking disease progression. When this assessment is underestimated, there may be an increased risk of complications, which can lead to greater morbidity and mortality among asthma patients.^23^^,^^24^

With the increasing use of digital technologies, important issues related to data security and privacy emerge, especially in the pediatric context. The collection and storage of sensitive health data require robust protection mechanisms to prevent security breaches. The literature warns that the trust of patients and caregivers in the use of these technologies may be compromised if these concerns are not adequately addressed.^19^^,^^25^

Although international recommendations aim for complete control of asthma symptoms, achieving this goal is difficult, in part due to limitations in patient assessment.^26^^,^^27^ Studies show that there is a discrepancy between doctors and patients regarding the state of asthma control, and many patients underestimate the severity of their symptoms, which can lead to inadequate treatments.^28^

It is also essential to consider the temporal trends of hospitalizations and deaths due to asthma in Brazil, especially among children and adolescents. Despite advances in prevention and treatment strategies, hospitalization and mortality rates from asthma still pose a significant challenge, highlighting the ongoing need for effective and accessible interventions.^29^ In this context, the integration of advanced technologies, such as artificial intelligence (AI), could play a crucial role in improving clinical outcomes. AI has the potential to revolutionize pediatrics by providing powerful tools for personalizing treatment and predicting complications, which can be particularly beneficial in managing chronic diseases like asthma.^30^ The combination of these technological approaches with preventive strategies could thus represent a significant advancement in reducing morbidity and mortality rates associated with asthma in pediatric populations.

Despite the progress observed, this study presents some limitations that warrant further discussion. Firstly, the small number of articles analyzed limits the scope of the findings and may affect the representativeness of the conclusions. This constraint also impacts the generalizability of the results to broader populations. Additionally, there was a predominance of older individuals among the study participants. This may introduce bias, as treatment responses and factors associated with asthma control can vary significantly across different age groups. Finally, the exclusive use of the ACT as a criterion for improvement represents another limitation. While the ACT is a widely recognized and validated tool, it does not account for other clinical and functional parameters that could provide a more comprehensive assessment of the patient's health status, such as objective measures of lung function or inflammatory biomarkers.

Moreover, the studies reviewed also demonstrate that digital tools, such as mobile applications, have the potential to improve adherence to asthma treatment through features like symptom tracking, medication reminders, and patient education. These tools empower patients and caregivers to take an active role in disease management, which can lead to improved clinical outcomes. However, the effectiveness of these technologies may depend on factors such as age, familiarity with technology, and the severity of asthma. For instance, older adults might face challenges with digital literacy, while younger users may find these tools more intuitive and accessible. Recognizing these differences is crucial to ensuring that digital interventions are tailored to meet the needs of diverse patient populations.

It is suggested for future research aimed at developing technologies like those evaluated in this study to consider data security and privacy when developing and implementing new digital tools for asthma management, as well as to focus on the aspect of individual personalization.

This systematic review and meta-analysis demonstrated that support tools based on web technologies and mobile devices have the potential to significantly improve the management of pediatric asthma. The studies analyzed indicate that these technologies can enhance asthma control, as reflected in improvements in ACT scores.

Despite the limitations found, such as variability in study designs and lack of standardization in outcome measures, the findings are promising.

However, to build a more robust and comprehensive evidence base, more research is needed, especially randomized controlled trials with greater standardization of outcome measures. These future investigations will be essential to inform clinical practice and the formulation of effective health policies in the management of pediatric asthma.

## Funding

None.

## Conflicts of interest

The authors declare no conflicts of interest.
